# Reply to the letter to the editor regarding the study “The effectiveness of gabapentin and gabapentin/montelukast combination compared with dextromethorphan in the improvement of COVID‐19‐related cough: A randomized, controlled clinical trial”

**DOI:** 10.1111/crj.13619

**Published:** 2023-04-26

**Authors:** Atousa Hakamifard, Rasool Soltani

**Affiliations:** ^1^ Department of Infectious Diseases, School of Medicine Isfahan University of Medical Sciences Isfahan Iran; ^2^ Infectious Diseases and Tropical Medicine Research Center Shahid Beheshti University of Medical Sciences Tehran Iran; ^3^ Infectious Diseases and Tropical Medicine Research Center Isfahan University of Medical Sciences Isfahan Iran; ^4^ Department of Clinical Pharmacy and Pharmacy Practice, School of Pharmacy and Pharmaceutical Sciences Isfahan University of Medical Sciences Isfahan Iran

We thank the authors of the letter to the editor for their careful reading of our paper entitled “The effectiveness of gabapentin and gabapentin/montelukast combination compared with dextromethorphan in the improvement of COVID‐19‐related cough: A randomized, controlled clinical trial” that was registered at the Iranian Registry of Clinical Trials, registration number: IRCT20150721023282N24.^1^

We would like to discuss the issues raised by the authors of the letter and to provide further information.

In this trial study, while considering that the dextromethorphan in the control group was in the form of syrup and considering that gabapentin and montelukast do not have the syrup form, it was not possible to prepare all the three drugs in the form of syrup, and as a result, it was not possible to conduct the study in blind format. Therefore, despite the ideality of blinding the study, it was not possible, and the study was conducted in an open‐label format. Also, we were aware of the risk of bias in the study, and we do not deny it.

In the case of adverse effects, we did not ask about specific potential side effects, but that we collected patients' spontaneous reports of side effects. Due to the doses consumed and the short period of 5 days in the study, we did not have any reports of side effects from the patients.

In the case of smoking history, it is correct and should have been recorded in the baseline data, but in the case of covid treatment, because patients who had chronic respiratory disease or allergy, and had oral intake intolerance, were all excluded from the study, all patients received the same type of treatment. In addition, the criterion for entering patients was cough with a Breathlessness, Cough, and Sputum Scale (BCSS) score of at least 2 based on its cough subscale, which covers the difference in the underlying disorder causing cough.

In the present study, both experimental interventions gabapentin and gabapentin/montelukast groups showed significant reduction in the severity of COVID‐19‐induced cough; however, the observed effects was significantly less than dextromethorphan as a standard antitussive drug. Furthermore, the combination of gabapentin/montelukast showed more improvement in cough compared with gabapentin alone. Cough reduction was significant in all three groups. This study was a non‐inferiority trial, and it was shown that gabapentin and gabapentin/montelukast were at least as effective as dextromethorphan in reducing cough and overall, according to our results, both drugs could be appropriate alternatives for dextromethorphan for improvement of cough in patients with COVID‐19.

## CONFLICT OF INTEREST STATEMENT

None.

## Data Availability

The data that support the findings of this study are available on request from the corresponding author. The data are not publicly available due to privacy or ethical restrictions.
